# Comparison of plasma cell bone marrow counts by different methods in patients diagnosed with plasma cell disorders

**DOI:** 10.1016/j.htct.2024.06.002

**Published:** 2024-08-21

**Authors:** Claudia Monteiro, Paulo Campregher, Denise Pasqualin, Nydia Bacal, Liliana Suganuma, Elvira Velloso

**Affiliations:** Hospital Israelita Albert Einstein, São Paulo, SP, Brazil

**Keywords:** Multiple myeloma, Bone marrow aspirate, Flow cytometry, Bone marrow biopsy, Plasma cell infiltration

## Abstract

**Introduction:**

Plasma cell quantification in bone marrow is important for diagnosis, prognosis, and treatment of plasma cell diseases. It can be performed by several methods such as aspiration, imprint and flow cytometry, and biopsy.

**Objectives:**

To compare plasma cell counts at diagnosis of plasma cell diseases using different methods.

**Methods:**

An observational study was carried out of laboratory results of adult patients with plasma cell diseases, who underwent aspiration, imprint cytology, flow cytometry (CD38, C138) and biopsy in a single institution between January 2015 and May 2021. The intraclass correlation coefficient was used to assess agreement between different methods with results stratified into three groups: <10%; 10–59% and ≥60% of infiltration.

**Results:**

Sixty-seven cases were studied: 59.7% were men with a median age of 70 (range: 32–85) years. The diagnoses were multiple myeloma in 61%, gammopathy of undetermined significance in 25.4%, smoldering myeloma in 6% and other plasma cell dyscrasias in 7.6%. Less than 10% infiltration was found in 32 (47.7%), 35 (52.2%), 44 (65.7%) and 25 (37.3%) of patients, respectively by aspiration, imprint cytology, flow cytometry and biopsy. Infiltration ≥60% was detected in 7 (10.4%), 4 (6.0%), 2 (3.0%) and 21 (31.3%) cases, respectively. There was disagreement between the results in 37 (55.2%) of patients. Of these, 28 had greater infiltration in biopsies. The concordance (Kappa index) of biopsy with aspiration, imprint and flow cytometry was 0.501, 0.408 and 0.17; of aspiration with imprint and flow cytometry, it was 0.738 and 0.541 and between imprint and flow cytometry, it was 0.573%.

**Conclusions:**

Only aspiration and imprint cytology results agreed. Biopsy showed greater infiltrations than the other methods, but aspiration, and imprint and flow cytometry provided additional data in the diagnosis and thus should also be performed.

## Introduction

Plasma cell disorders are a heterogeneous group of diseases characterized by abnormal proliferation and function of plasma cells with frequent detection of a monoclonal paraprotein in the serum, urine, bone marrow or in other tissues.^1^

Multiple myeloma (MM) holds particular significance among these disorders, comprising approximately 1% of all cancer cases.^2^ Moreover, it ranks as the second most prevalent hematological malignancy in high-income countries.^3^ The diagnosis of MM requires the identification of one or more myeloma defining events, together with evidence demonstrating either 10% or more clonal plasma cells in bone marrow examinations or confirmation of a biopsy-proven plasmacytoma. Myeloma defining events consist of hypercalcemia, renal failure, anemia, lytic bone lesion features and these three specific biomarkers: clonal bone marrow plasma cells ≥60%, involved serum free light chain level ≥100 mg/L, and more than one focal lesion on magnetic resonance images.^2^

New treatments such as immunomodulatory drugs, proteasome inhibitors, and CD38-targeted antibodies have extended survival, but ultimately, most patients will die from their disease with others dying from treatment-related complications.^3^

MM is usually preceded by monoclonal gammopathy of undetermined significance or smoldering myeloma, both asymptomatic conditions where treatment is typically not indicated. Therefore, a detailed diagnostic investigation of clinical features, and radiologic and laboratory evaluations are needed to differentiate between MM, which requires treatment, and other conditions.^3^ Table 1 outlines the diagnostic criteria of different plasma cell diseases.

**Table 1:** Diagnostic criteria of other plasma cell diseases.

Non-IgM monoclonal gammopathy of undetermined significance (MGUS)	Smoldering multiple myeloma	Solitary plasmacytoma
Serum monoclonal protein (non-IgM type) <3 g/dL Clonal bone marrow plasma cells <10% Absence of end-organ damage that can be attributed to a plasma cell proliferative disorder.	Serum monoclonal protein (IgG or IgA) ≥3 g/dL, or urinary monoclonal protein ≥500 mg/24 h or clonal bone marrow plasma cells 10–60% and Absence of myeloma defining events or amyloidosis	Biopsy-proven solitary lesion of bone or soft tissue with evidence of clonal plasma cells Normal bone marrow with no evidence of clonal plasma cells Normal skeletal investigation and magnetic resonance imaging or computed tomography results of spine and pelvis (except for the primary solitary lesion) Absence of end-organ damage that can be attributed to a lymphoplasma cell proliferative disorder

Adapted from Rajkumar et al.^2^

According to European hematology Association-European Society for Medical Oncology (EHA-ESMO)^4^ and National Comprehensive Cancer Network (NCCN)^5^ guidelines, the initial workup of MM involves a bone marrow evaluation with immunohistochemistry and/or flow cytometry to confirm plasmacytosis and monoclonality. This assessment can be performed by three different methods: Bone marrow aspiration, bone marrow imprint, or bone marrow biopsy.^6^

BMA is an easy, reliable, and fast method of marrow evaluation. It provides information about the numerical and cytological features of marrow cells. The sample obtained can also be used for further examination using cytogenetics, and molecular and flow cytometric methods (7).

BMB offers a comprehensive insight into the spatial interrelationships of cellular components and the broader architectural framework of the bone marrow. This procedure proves invaluable in assessing cellular composition and investigating potential focal lesions and bone marrow fibrosis. However, it requires a sequence of time-intensive procedures, encompassing fixation, decalcification, dehydration, and block preparation of samples leading to significant delays in the acquisition of results.^7^^,^^8^

BMI also provides a good cytological evaluation and cellularity quantification and its assessment is faster than that of BMB^8^

Furthermore, flow cytometry has the advantage of distinguishing immunophenotypic features between normal and clonal plasma cells.^9^

Currently these methods are perceived as complementary. However, BMB is more invasive, associated with pain and with longer turnaround time.^7^ In addition, with the improvement of laboratory techniques over the years and with the association of methods, it is possible that some of the limitations of BMA have been overcome, improving its correlation with BMB and BMI.

Some studies have been conducted comparing different bone marrow examination techniques in the MM workup, but all were concluded over five years ago, and none evaluated the Brazilian population.^7^^,^^10^, ^11^, ^12^

Therefore, this study aims to compare plasma cell counts of patients with plasma cell disorders evaluated at Hospital Israelita Albert Einstein (HIAE) to determine the correlation between four different methods: BMA, BMI, BMB, and FC.

## Material and methods

A descriptive observational study was carried out of laboratory results of diagnostic examinations of patients with plasma cell diseases at HIAE between January 2015 and May 2021. Only adult patients whose samples were obtained from bone marrow were included. Individuals who had not preformed all of the following tests were excluded: BMA, BMI, FC and BMB. Data were obtained from the electronic medical records of patients after approval of the study by the institutional ethics committee.

BMAs were performed by puncture of the posterior superior iliac crest with an Illinois needle. After staining the smear obtained, the percentage of plasma cells was defined by morphological analysis and differential counting of 250 cells by two experienced hematologists.

BMBs were performed with a Jamshidi needle at the same puncture site as the BMA. The immunohistochemical panel used identified the expressions of CD138, CD56, CD20, Kappa and Lambda antibodies. Plasma cell estimations were performed by a pathologist specialized in hematology after observing the cells expressing CD138. The result is expressed as a percentage of bone marrow affected by plasma cell infiltration.

The BMIs were prepared by rolling the biopsy fragment between two slides, which resulted in an imprint of the fragment. Subsequently, the slides were stained and two experienced hematologists quantified the percentage of plasma cells by the differential counting of 100 cells employing morphological analysis.

FC was performed using a BD FACSCanto TM II flow cytometer – Becton Dickinson. Two tubes were used to identify the plasmacytes according to the antibodies, fluorochromes and clones. Tube 1: CD38 (FITC, T16), CD56 (PE, N901 – NKH-1), CD20 (PERCP, 2H7), CD19 (PC7, J3-119), intracytoplasmic Kappa (APC, TB28-2), intracytoplasmic Lambda (APC—H7 1-155-2), CD45 (V450, 2D1), CD138 (V500, MI15) and tube 2: CD38(FITC, T16), CD28(PE, L293), CD27 (PERCP-Cy5.5, L128), CD19 (PC7,J3-119), CD117 (APC, 104D2), CD81 (APC—H7, JS-81), CD45(V450, 2D1), CD138 (V500, MI15). A total of 1.7 million events was acquired. Analyzes were performed using the Infinicity™ 2.0 - Cytognos software. The analysis reports were read by analysts and hematologists with expertise in FC.

After quantification of plasma cell percentages in the BMA, BMI, FC and BMB, the results were stratified into three distinct categories based on the extent of cellular infiltration: below 10%, between 10% and 59%, and 60% or higher. Subsequently, a comparative assessment of the different methodologies was conducted, followed by an analysis of the concordance between these outcomes.

The Kappa test was used with the Statistical Package for Social Sciences (SPSS: version 1.0.0.1406). Data are presented as medians and ranges for numerical variables and absolute values and percentages for categorical variables.

## Results

Initially 106 patients were enrolled in this study however, as only 67 had performed all four tests at the same time, the analysis included only 67 cases.

### Clinical and demographic characteristics

The patients studied were aged between 32 and 85 years, with most cases in the 8th decade of life. The ratio of men to women was 1.48:1. The most frequent diagnosis was MM and the least frequent were light chain deposition disease, solitary plasmacytoma, and plasma cell leukemia. Most patients had IgG Kappa paraprotein and standard risk, as shown in Table 2.

**Table 2:** Clinic and demographic characteristics of the studied population.

Characteristic		n (%)	Median (Range)
Age			70 (32–85)
Sex			
	Female	27 (40.3)	
	Male	40 (59.7)	
Diagnosis			
	MGUS	17 (25.4)	
	Smoldering myeloma	4 (6.0)	
	Multiple myeloma	41 (61.0)	
	Plasma cell leukemia	1 (1.5)	
	Solitary plasmacytoma	1 (1.5)	
	Light chain deposition disease	1 (1.5)	
	AL amyloidosis	2 (3.0)	
Paraprotein	IgG Kappa IgG Lambda IgA Kappa	26 (38.9) 7 (10.4) 9 (13.4)	
	IgA Lambda IgM Kappa Kappa Lambda Unknown	4 (6.0) 1 (1.5) 7 (10.4) 6 (9.0) 7 (10.4)	
FISH	Standard risk High risk Double hit	43 (64.0) 20 (30.0) 4 (6.0)	
Year of diagnosis			
	2015	3 (4.5)	
	2016	13 (19.5)	
	2017	9 (13.4)	
	2018	11 (16.4)	
	2019	11 (16.4)	
	2020	11 (16.4)	
	2021	9 (13.4)	
Total		67 (100)	

MGUS: Monoclonal gammopathy of undetermined significance; AL amyloidosis: amyloid light-chain amyloidosis; FISH: Fluorescence in situ hybridization (FISH).

### Plasma cell infiltration

The percentages of infiltration identified by the four methods are shown in Table 3.

**Table 3:** Percentage of infiltration according to the method.

Method	<10%	10–59%	≥60%
Bone marrow aspiration	32	28	7
Bone marrow imprint	35	28	4
Flow cytometry	44	21	2
Bone marrow biopsy	25	21	21

### Agreement between the different methods

A comparison of the methods was made as shown in Table 4, Table 5, Table 6. In general, there was no satisfactory agreement between methods, except for between BMA and BMI. In most cases, a greater number of plasma cells were identified by BMB.

**Table 4:** Agreement of plasma cell infiltration of bone marrow biopsy and the other methods.

	BMB								Kappa	95% confidence interval
Variable	0–9		10–59		60 or +		Total			
	n	%	n	%	n	%	n	%		
**BMA**									0.501	0.344–0.658
0–9	24	35.8	7	10.4	1	1.5	32	47.8		
10–59	1	1.5	14	20.9	13	19.4	28	41.8		
60 or +	0	0.0	0	0.0	7	10.4	7	10.4		
**BMI**									0.408	0.255–0.561
0–9	25	37.3	9	13.4	1	1.5	35	52.2		
10–59	0	0.0	12	17.9	16	23.9	28	41.8		
60 or +	0	0.0	0	0.0	4	6.0	4	6.0		
**FC**									0.170	0.033–0.307
0–9	24	35.8	16	23.9	4	6.0	44	65.7		
10–59	1	1.5	5	7.5	15	22.4	21	31.3		
60 or +	0	0.0	0	0.0	2	3.0	2	3.0		
**Total**	**25**	**37,3**	**21**	**31,3**	**21**	**31,3**	**67**	**100**		

BMB: bone marrow biopsy. BMA: bone marrow aspirate. BMI: bone marrow imprint. FC: flow cytometry.

**Table 5:** Agreement of plasma cell infiltration of bone marrow aspirate and the other methods.

	BMA								Kappa	95% confidence interval
	0–9		10–59		60 or +		Total			
Variable	n	%	n	%	n	%	n	%		
**BMI**									0.738	0.595–0.881
0–9	31	46.3	4	6.0	0	0.0	35	52.2		
10–59	1	1.5	23	34.3	4	6.0	28	41.8		
60 or +	0	0,0	1	1.5	3	4.5	4	6.0		
**FC**									0.541	0.370–0.712
0–9	32	47.8	12	17.9	0	0,0	44	65.7		
10–59	0	0.0	16	23.9	5	7.5	21	31.3		
60 or +	0	0.0	0	0.0	2	3.0	2	3.0		
**Total**	**32**	**47,8**	**28**	**41,8**	**7**	**10,4**	**67**	**100**		

BMA: bone marrow aspirate. BMI: bone marrow imprint. FC: flow cytometry.

**Table 6:** Agreement of plasma cell infiltration of bone marrow imprint and the other methods.

	BMI								Kappa	95% confidence interval
	0–9		10–59		60 or +		Total			
Variable	N	%	n	%	n	%	n	%		
**FC**									0.573	0.399–0.747
0–9	34	50.7	10	14.9	0	0.0	44	65.7		
10–59	1	1.5	17	25.4	3	4.5	21	31.3		
60 or +	0	0.0	1	1.5	1	1.5	2	3.0		
**Total**	**35**	**52.2**	**28**	**41.8**	**4**	**6,0**	**67**	**100**		

BMI: bone marrow imprint. FC: flow cytometry.

### Discrepancies between methods

**Figure 1:**
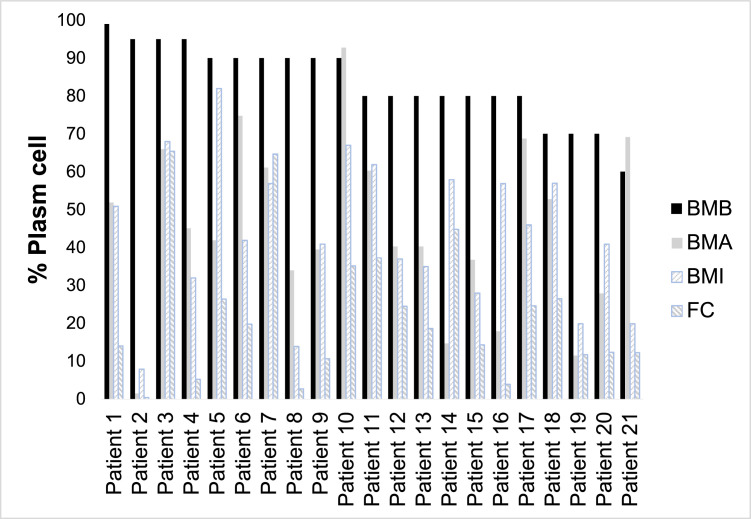
Percentage of plasma cells detected by bone marrow biopsy (BMB), bone marrow aspirate (BMA), bone marrow imprint (BMI) and in flow cytometry (FC) for the 21 patients with more than 60% of plasma cells detected by BMB.

Thirty-seven of 67 cases (55.2%) showed a difference in the percentage of plasma cells found between the methods performed, that is, at least one of the tests presented a result classified in a different group from the others. Of these, eight showed a discrepancy only in the result of FC. Of the remaining 29, 28 had a higher percentage of plasma cells detected in BMB, 12 had a description of contamination with peripheral blood in the BMA, 14 had moderate to severe bone marrow fibrosis, and one had a nodular infiltration of plasma cells. In one case, the BMB had an inadequate sample. Considering the BMB as the gold standard for plasma cell counts, it was observed that the greatest disagreement occurs in the plasma cell count range greater than 60%. Figure 1 shows plasma cell counts using the different methodologies for the 21 cases with >60% plasma cells detected in bone marrow biopsy.

## Discussion

The quantification of plasma cells is important to define the diagnosis of plasma cell dyscrasias and for this it should be performed in the initial workup.^12^ The methods used differ from each other in terms of the discomfort of the patient, the time taken to release the result and additional information provided, such as morphological characteristics by BMA and degree of fibrosis by BMB.

In the current study, higher plasma cell infiltration levels were found ​​by BMB, and the agreement in the quantification of these cells was weak to moderate between the methods except between the BMA and BMI. Therefore, the BMB report can change the diagnosis and consequently the prescription of therapy, and should be performed in all patients at the time of diagnosis. This test also allows a better comparison of infiltration during patient follow-up. This is important since the percentage of plasma cells in the bone marrow before autologous transplantation has prognostic value in MM.^13^

FC had the lowest concordance with the other methods. This may be because it tends to underestimate the number of plasma cells when compared to morphological analysis. There are several reasons for this, such as the hemodilution of samples used in immunophenotyping compared to samples collected for a BMA smear and the different distributions of plasmacytes in lipid-rich particles on bone marrow aspirate slides compared to the distribution in the lipid-depleted sample for immunophenotyping. In addition, because these cells are more susceptible to mechanical damage, some are lost during processing, antibody labeling, and acquisition for FC.^9^ Despite this, this method is crucial in determining monoclonality in plasma cell diseases and for minimal residual disease monitoring.

Moreover, potential explanations for the observed low concordance among the alternative exams lie in the inherent nature of plasma cell disorders, particularly MM. These diseases exhibit focal engagement characterized by nodular infiltration patterns, posing challenges for their accurate identification in scattering or touch-based analyses. Additionally, the diseases commonly evolve alongside degrees of moderate to severe fibrosis, a factor that can render diagnoses unattainable by techniques incapable of depicting tissue architecture.^9^^,^^11^^,^^12^ Another reason for the discrepancy in results between the BMA and the other methods is sample contamination with peripheral blood, which causes dilution and reduces the concentration of plasma cells.

However, although the BMB was more reliable to show infiltration for most patients, this did not occur in one of the cases of this study, in which the number of plasma cells seen in the BMA was greater than in the BMB. This was due to an inadequate sample for histopathological analysis. In this case, BMA was essential for plasma cell quantification, and FC for demonstrating clonality.

Results from the BMA, BMI, and FC methods offer a quicker turnaround time, enabling fast initiation of treatment upon confirmation of diagnosis. Additionally, these methods can provide supplementary insights; BMA and BMI can give information about morphological characteristics, while FC can shed light on immunophenotypic expression. Given these advantages, it is advisable to conduct these tests at the point of diagnosis whenever feasible.

It is worth noting that this study had a few limitations. Firstly, it was a retrospective study conducted in a single center. Additionally, the tests relied on techniques that underwent slight changes over time. Moreover, the study had a small number of participants as some patients only completed certain tests at the institution's laboratory, resulting in their exclusion from the study.

## Conclusion

BMB presents higher plasma cell counts in plasma cell disorders when compared to other methods, which can change the therapeutic approach for some patients, so it should always be performed at diagnosis. In addition, in our experience, BMA, BMI, and FC are complementary to the histopathological findings, especially when the biopsy sample is inadequate.

## Conflicts of interest

The author declares no conflicts of interest..
